# Trends and challenges of multi-drug resistance in childhood tuberculosis

**DOI:** 10.3389/fcimb.2023.1183590

**Published:** 2023-06-02

**Authors:** Zengfang Zhuang, Lin Sun, Xiaorui Song, Hanzhao Zhu, Lianju Li, Xintong Zhou, Kaixia Mi

**Affiliations:** ^1^ Chinese Academy of Sciences (CAS) Key Laboratory of Pathogen Microbiology and Immunology, Institute of Microbiology, Chinese Academy of Sciences, Beijing, China; ^2^ Savaid Medical School, University of Chinese Academy of Sciences, Beijing, China; ^3^ Beijing Children’s Hospital, Capital Medical University, Beijing, China; ^4^ Henan International Joint Laboratory of Children’s Infectious Diseases, Children’s Hospital Affiliated to Zhengzhou University, Henan Children’s Hospital, Zhengzhou Children’s Hospital, Zhengzhou, China; ^5^ School of Basic Medicine, Shandong First Medical University and Shandong Academy of Medical Sciences, Jinan, China

**Keywords:** multidrug resistant, childhood tuberculosis, diagnostic, treatment, epidemiology

## Abstract

Drug-resistant tuberculosis (DR-TB) in children is a growing global health concern, This review provides an overview of the current epidemiology of childhood TB and DR-TB, including prevalence, incidence, and mortality. We discuss the challenges in diagnosing TB and DR-TB in children and the limitations of current diagnostic tools. We summarize the challenges associated with treating multi-drug resistance TB in childhood, including limitations of current treatment options, drug adverse effects, prolonged regimens, and managing and monitoring during treatment. We highlight the urgent need for improved diagnosis and treatment of DR-TB in children. The treatment of children with multidrug-resistant tuberculosis will be expanded to include the evaluation of new drugs or new combinations of drugs. Basic research is needed to support the technological development of biomarkers to assess the phase of therapy, as well as the urgent need for improved diagnostic and treatment options.

## 1 Introduction

Tuberculosis (TB) is a major infectious disease that seriously threatens millions of people worldwide, including children. An estimated 10.6 million new cases and 1.4 million deaths were reported in 2021 alone (World Health Organization, 2022), while cases of TB in children account for 11% of all TB cases (1.2 million out of 10.6 million) and 14% of TB deaths (0.2 million out of 1.413 million).

Drug-resistant TB (DR-TB) is a form of TB that is resistant to the most commonly first-line anti-TB drugs, making it more difficult to treat. The WHO divides DR-TB into five categories: isoniazid-resistant TB, rifampicin-resistant (RR) TB, multi-drug resistant (MDR) TB (TB resistant to isoniazid and rifampicin), pre-extensively drug resistant (preXDR) TB, which is MDR-TB with resistance to a fluoroquinolone, and extensively drug resistant (XDR) TB, which is TB resistant to rifampicin plus any fluoroquinolone plus at least one other priority A drug (bedaquiline or linezolid). DR-TB is a significant threat to public health, as it is more difficult and expensive to treat than drug-susceptible (DS) TB, and is associated with higher rates of treatment failure, relapse, and mortality.

Children with DR-TB face more unique challenges. Childhood TB was neglected in the first decade of the 21st century - children were not recognized as contributing to the TB epidemic and were therefore not prioritized in the global TB response. It was not until 2012 that the WHO began reporting pediatric (<15 years) TB estimates. After years of neglect (Sandgren et al., 2012), the detection methods are inadequate and the diagnosis of pediatric cases of MDR-TB is a challenge. Of the estimated 1.1 million children who develop TB, only 399, 000 (36.5%) were identified by National Tuberculosis Programs (NTPs) in 2020 (World Health Organization, 2022). Below 5 years old, there are only 27.5% of children are identified (World Health Organization, 2021a; World Health Organization, 2022). The detection rate of multi-drug resistant TB in children is low, due to the difficulty in diagnosing TB. Despite the combination therapy is available for children TB, but not based on clinical trial evidence. The biomarkers to monitor treatment response during TB therapy are urgently needed. The basic research should be a strength to develop diagnosis and treatment against children TB, especially for MDR- children TB. In this review, we summarize the progress of mycobacterial disease diagnosis and the treatment of MDR-TB in children.

## 2 Epidemiology

Childhood TB is a major public health concern, particularly in low- and middle-income countries, where poverty, malnutrition, and overcrowding contribute to the spread of the disease. The incidence rate of childhood tuberculosis varies widely across different regions of the world, with the highest rates observed in sub-Saharan Africa and Southeast Asia. In 2019, there were 465,000 patients with MDR/RR-TB, with a treatment success rate of 59%. About 180,000 people died from MDR/RR-TB in 2019 (Dean et al., 2022). In 2020, about 132,222 new RR-TB patients were identified, of which about 25% were MDR-TB (World Health Organization, 2022).

According to World Health Organization (WHO), an estimated 1 million children develop TB each year. Children account for 12% of the global TB burden (1.2 million out of 10 million) and 16% of TB deaths (0.20 million out of 1.28 million in 2020). In addition, approximately 25,000-32,000 children develop MDR-TB each year (Jenkins et al., 2014; Dodd et al., 2016; Jenkins and Yuen, 2018), only 3-4% of pediatric MDR-TB are diagnosed and treated, and 21% of children with MDR-TB may die as a result (Jenkins and Yuen, 2018).

TB and MDR-TB in children remain to be major public health challenges worldwide. The burden of MDR-TB in children is highest in settings with high levels of MDR-TB in adults, and MDR-TB is associated with poorer treatment outcomes than DS-TB. MDR-TB also has worse treatment outcomes than DS-TB. Therefore, there is an urgent need for improved strategies to prevent, diagnose, and treat TB and MDR-TB in children.

## 3 Diagnostic challenges of drug-resistant tuberculosis in children

The serials review has summarized the mechanisms of mycobacterial drug tolerance and resistance (Ramaswamy and Musser, 1998; Singh and Kaur, 2011; Walker et al., 2022). The tolerance and resistance mechanisms include molecular mutations (Walker et al., 2022), epistasis (Borrell et al., 2013; Wong, 2017), DNA epigenetics, and tolerance (Gagneux et al., 2006; McBryde et al., 2017).

The development of new diagnostic tools is needed, that quickly and accurately identify drug resistance in mycobacteria, which will aid for the effective treatment of MDR-TB. Diagnosing TB and DR-TB in children presents several challenges due to the limitations of current diagnostic tools. The current gold standard for diagnosing TB is sputum culture, which can take several weeks to yield results. Children with TB, who harbor small amounts of *Mycobacterium tuberculosis* (MTB), have lower sensitivity to current diagnostic tests. Primary pulmonary TB is more common in children with TB, and the clinical symptoms and signs are often non-specific and other diagnostic tests, such as X-rays and the tuberculin skin test, are easily misdiagnosed and missed, particularly in areas with high rates of tuberculosis and HIV co-infection. Additionally, these tests are unable to differentiate between DR and DS-TB strains, making it challenging to identify patients who require alternative treatments. In addition, an estimated 69% of missed cases occurred among children under 5 years of age, with a low case detection rate relative to other age groups. This is mainly due to the characteristics of children’s growth and pathogenesis. Therefore, accurate and accessible point-of-care tests (POCTs) are needed to detect TB in children to reduce TB-related morbidity and mortality in this vulnerable population (Mukherjee et al., 2011; Graham et al., 2015; Reuter et al., 2019; Nicol and Zar, 2020; World Health Organization, 2022).

In recent years, a variety of POCTs for TB have been developed, such as the molecular detection of MTB using loop-mediated isothermal amplification (LAMP) (Mitarai et al., 2011) and the portable polymerase chain reaction (PCR)-based GeneXpert. (Lawn, 2015; Bloom et al., 2017; World Health Organization, 2021a; World Health Organization, 2021b).

According to the 2021 Global Tuberculosis Report, WHO recommended some new diagnostic tests for non-invasive samples like sputum, gastric aspirate, or stool by Xpert MTB/RIF Ultra assay. (WHO consolidated guidelines on tuberculosis Module 5, 2022). The diagnostic of Xpert MTB/RIF Ultra assay was recommended for the initial diagnostic test and the detection of rifampicin resistance (Bloom et al., 2017). According to Kay et al., Xpert Ultra has varying sensitivity to different specimen types. Sputum has the highest sensitivity, followed by gastric aspirate and stool. Nasopharyngeal aspirate has the lowest sensitivity (Kay et al., 2022). A study examines Xpert Ultra on stool to diagnose PTB in children found that it^’^s sensitivity and specificity were 58.6% and 88.1%, respectively, while Xpert on stool had its sensitivity of 37.9% and a specificity of 100.0% (Kabir et al., 2021). However, this test does not always detect the mycobacterial infection accurately, especially in children with severe acute malnutrition (SAM), or those with HIV.

The whole-genome sequencing (WGS) is recommended as a valuable tool for the surveillance of DR-TB by WHO (World Health Organization, 2020). WGS can thoroughly detect all genes related to drug resistance, which can provide valuable information for clinical treatment, particularly for MDR-TB (Votintseva et al., 2017; Doyle et al., 2018). WGS has also been studied for the identification of MDR-TB in children (Zhang et al., 2021). Compared with the conventional phenotypic drug susceptibility test (DST), it provides accurate results for both first-line and second-line anti-TB drugs. Recently, Studies showed the method for sequencing DNA directly from sputum samples provides a promising approach to diagnosis (Votintseva et al., 2017; Doyle et al., 2018). WGS is a promising approach for predicting resistance to isoniazid, rifampin, pyrazinamide, levofloxacin, streptomycin, second-line injectable drugs (SLIDs) and prothionamide. It has satisfactory accuracy, with sensitivity and specificity of over 85.0% (Chen et al., 2019). For the detection of MDR-TB in children, sputum samples are still the limitation of the application, and improving the preparation of test specimens will provide potential solutions for the accurate detection of MDR-TB in children.

In addition, age is also a key consideration in the diagnosis of TB in children (Kay et al., 2022). This may be due to differences in immune status and response to MTB at different ages. When MTB infects the host lung, it causes host inflammation and tissue damage. Recent studies using combined multi-omics such as metabolomics, lipidomics, and cytokine profiling have shown that host metabolism plays a crucial role in driving inflammation against TB (Pitaloka et al., 2022; Yu et al., 2023). These studies have indicated the potential benefits of taking advantage of the host’s response for clinical diagnosis.

Metabolomics allows for the quantitative profiling of both the infected host and MTB, enabling the identification of biomarkers for diagnosing active TB, identifying latent TB infection (LTBI), predicting the risk of developing active TB, and monitoring the effectiveness of anti-TB drugs (Szewczyk et al., 2013; Salgado-Bustamante et al., 2018; Magdalena et al., 2022).

Mass spectrometry, gas chromatography-time-of-flight mass spectrometry (GC-TOF MS), ultra-performance liquid chromatography-mass spectrometry (UPLC-MS), or nuclear magnetic resonance spectroscopy are platforms used in metabolomics to characterize metabolites as biomarkers from biological samples such as blood, urine, cerebrospinal fluid (CSF), fecal wastes and breath (Lau et al., 2015; Isa et al., 2018). Biomarkers have been shown to identify DR strains of MTB. A study compared the lipid profiles of drug-sensitive (DS) and DR strains of MTB and found differences in fatty acyls and glycerophospholipids that could potentially be used as biomarkers for identifying drug resistance (Pal et al., 2017). Rego et al. found DS, MDR, and extensively drug-resistant (XDR) strains of TB had different profiles, which could be used to predict their susceptibility or resistance to drugs (Rego et al., 2021). Some other non-sputum diagnostics are also promising for improved pediatric MTB diagnosis. The urine-based lipoarabinomannan (LAM) assay has been endorsed by WHO for TB diagnosis as sensitivity increases significantly in patients with lower CD4 cell counts. The value of urine-based lipoarabinomannan (LAM) antigen tests for diagnosing tuberculosis in children has been shown to vary depending on test used. The sensitivity and specificity of the *Mycobacterium. tuberculosis* enzyme-linked immunosorbent assay (MTB-LAMELISA), Alere Determine TB LAM Ag (Alere LAM) test, and the Fuji LAM diagnostic procedure among pediatric cases below 15 years with TB were 16.0% and 95.61%; 45.90% and 80.42%; and 52.32% and 89.37%, respectively (Seid et al., 2022). Blood transcriptional markers and cell-free DNA in urine are promising additional candidates for non-sputum triage or confirmatory TB tests (Turner et al., 2020; Hiza et al., 2021). These tests have the potential to improve the accuracy and speed of MDR-TB diagnosis, particularly in cases where sputum samples are difficult to obtain or analyze. The sensitivity and specificity of Cepheid’s Xpert MTB-HR fingerstick blood test using 3-host mRNA, were 90% and 55.8%, respectively, regardless of HIV status (Sutherland et al., 2022). CD38-based TAM-TB assay is also a sputum-independent, blood sample test that has been shown to have a specificity of 93.4% and a sensitivity of 82.2% (Hiza et al., 2021). As to children, both TAM-TB blood test and MTB-HR fingerstick blood test has been shown to be a reliable and highly specific tool for diagnosing TB in children, which is designed to detect progression from LTBI to active MTB (Hiza et al., 2021; Sutherland et al., 2022). Blood specimens can be used to diagnose children, but biomarkers specific to DR-TB need to be sought.

Other specimen types are also evaluated for TB test suitability. Gastric aspirates can be useful for children and the best way to obtain specimens from children less than 1 year of age. Xpert Ultra pooled sensitivity was 67.3% and 71.5% for children aged 1-4 years (Kay et al., 2022). For stool samples from children less than 1 year of age, Xpert Ultra pooled sensitivity was 65.2% and 43.3% for children 1-4 years. Samples that are more invasive like gastric aspirate show more accuracy with their high sensitivity (70.4%) and specificity (94.1) for children under one year. The WHO recommends that by 2021, the use of artificial intelligence (AI), and computer-aided detection for diagnosis based on X-ray databases in adults, but not in children (Codlin et al., 2021; Qin et al., 2021). As technology develops, AI should also be used to diagnose children. However, there are still limited data available on metabolic alterations that occur in children with TB (Dutta et al., 2020; Comella-Del-Barrio et al., 2021).

## 4 Treatment challenges for DR-TB in children

The emergence of MDR strains of *M. tuberculosis* presents an added challenge in the battle against TB. The main challenge in treating MDR-TB in children is the lack of effective drugs. Many of the drugs used to treat TB are either ineffective or have toxic side effects in children. This limits the treatment options and can lead to prolonged treatment periods, which can be especially difficult for children. The treatment of MDR-TB in children follows the same general principles as in adults (Pecora et al., 2021; Bossu et al., 2023). The WHO recommends using injectable-free regimens for children and has classified drugs into three groups: Group A includes levofloxacin/moxifloxacin, bedaquiline, and linezolid; Group B includes clofazimine and cycloserine; and Group C includes delamanid, ethambutol, pyrazinamide, ethionamide, para-aminosalicylic acid, and amikacin (details are listed in **Table 1**).

**Table 1:** Treatment Regimen for Children with Multi-Drug and Rifampicin Resistant TB.

Treatment Program		Treatment Drugs		Dosage*	Duration of Treatment
Short-course treatment program	6 months full oral regimen	Bedaquiline, Pretomanid, Linezolid/Moxifloxacin		/	6 months
	9 months full oral regimen	Bedaquiline, Fluoroquinolones, Linezolid, Ethionamide, Pyrazinamide, Ethambutol, Isoniazid, Clofazimine		/	9 months
Long-course treatment program		Group A	Bedaquiline	>12 y and > 30 kg body weight: 400 mg daily x 2 wk followed by 200 mg M/W/F x 24 wk Data on dose in younger children not yet available	Long-term
			Linezolid	Weight-banded dose: 5-9 kg = 15 mg/kg OD 10–23 kg = 12 mg/kg OD >23 kg =10 mg/kg OD	
			Levofloxacin	15–20 mg/kg/d	
			Moxifloxacin	10–15 mg/kg/d	
		Group B	Clofazimine,	2-5 mg/kg/d. Weight-banded dose: 50 mg gel capsule: 5- < 10 kg: 1 caps every 2nd d; 10-20 kg:1 caps/d; > 20 kg: 2 caps/d; 100 mg gel capsule: 5- < 10 kg: 1 caps Mon/Wed/Fri; 10-20 kg: 1 caps every 2nd day; >20 kg 1 caps everyday	
		Cycloserine/Terizidone	15–20 mg/kg/d		
		Group C	Ethambutol	15–25 mg/kg/d	
		Delamanid	>12 y/>35 kg: 100 mg twice daily 6–12 y/20-34 kg: 50 mg/kg twice daily Data in younger children not yet available		
		Pyrazinamide	30–40 mg/kg/d		
		Amikacin	15–20 mg/kg IMI or IVI daily		
		Ethionamide /Prothionamide	15–20 mg/kg/d		
		Meropenem	20–40 mg/kg 8 hourly (IV)		
		Amoxicillin-clavulanate	80 mg/kg/d in 2–3 divided doses of amoxicillin component – always combine with a carbapenem (not effective on its own)		
		Para-aminosalicylic acid	150–200 mg/kg daily as single or divided dose		

(1) This table is intended to guide the development of individualized MDR-TB long-course regimens (MDR-TB short-course regimens are essentially standardized in composition.) The drugs in Group C are listed in descending order of the strength of evidence for their recommended use. (2) There is insufficient evidence of safety and efficacy for Bedaquiline use beyond 6 months and for use in pediatric patients under 6 years of age, so the use of Bedaquiline in these cases should follow the “over-indication” use protocol. (3) There is insufficient evidence of safety and efficacy for delamanid use beyond 6 months and in pediatric patients under 3 years of age, therefore, the use of delamanid in these cases should follow the “over-indication” use protocol.

* Dosage information adapted from (Schaaf, 2019) (World Health Organization, 2018),

The drug regimen used to treat drug-resistant tuberculosis (DR-TB) in children is often complex and lengthy, leading to several challenges. One of the most concerning issues is the side effects associated with the drugs used to treat DR-TB, such as nausea, vomiting, hearing loss, and liver damage. These side effects can be especially challenging for children, who may have a difficulty tolerating the medications they are taking (Seddon et al., 2012; Schaaf, 2019). Additionally, treating DR-TB in children can be prolonged, with regimens lasting up to two years depending on disease severity and drug resistance. This can be difficult for children and their families to adhere to. Children with MDR-TB require specialized care, including close monitoring and support from healthcare providers. This can be difficult to provide in resource-limited settings, where there may be a shortage of trained health workers and limited access to diagnostic tools (Bossu et al., 2023). Furthermore, children with DR-TB may face depression, stigma, social isolation and low self-esteem (Snow et al., 2020).

It is important to develop new therapeutic strategies against MTB (such as monoclonal antibodies, TB immunotherapy, which include cytokines, immune cells and immunomodulatory drugs) or combinations of drug cocktails (combining the use of bedaquiline and/or pretomanid regimens of drugs in the replacement of first-line anti-TB drugs) (Kak et al., 2018; Olin et al., 2018; Rao et al., 2019; Mi et al., 2021; Lu et al., 2022).

In general, most children with TB have less severe disease than adults. Shorter treatment regimens than those used for adults may be effective in treating children with TB, but the evidence is lacking. Shorter treatment regimens may result in lower costs for families and health services, less potential toxicity, less risk of drug interactions in HIV-infected children, and fewer problems with adherence to full treatment. Shorter, safe and effective treatment regimens for children with DS and DR-TB benefit children with TB and their families, and are key interventions to achieve the goals of the End TB Strategy and the child-related targets set at the 2018 United Nations General Assembly High-Level Meeting on TB. New evidence from a recently completed trial on shortening treatment duration for DS-TB in children and adolescents paves the way for new recommendations for shorter treatment courses for this group. The recent WHO consolidated guidelines on TB, Module 5, and the accompanying operational handbook, provide four new recommendations on the treatment of TB disease in children (WHO).

Gaps in the treatment of children with MDR/RR-TB include a lack of safe and effective treatments, and limited access to diagnosis. There is a significant lack of clinical trials of TB drugs in children due to ethical and practical challenges. Conduct clinical trials in children is difficult because of the need for parental consent, the limited number of eligible participants, and the difficulty in measuring outcomes. The regulatory agencies or governments need to develop clear guidelines and requirements for drug development in children, provide incentives to encourage clinical trials for children drugs and address ethical and practical challenges. More research is needed on how best to treat MDR-TB in children, including understanding the long-term effects of different treatments. Antibiotics and vaccination have played important roles in reducing TB, and understanding the function and status of immune cells after MTB infection will provide scientific guidance for immunotherapy of TB. However, the field of pediatric immunology has been limited by the difficulty of collecting samples from children. Systems immunology is a scientific field that combines high-throughput analysis technology, with comprehensive data analysis to study the immune system from a holistic perspective. Single-cell sequencing technology can be used to obtain more comprehensive immunological information from small amounts of blood samples, which could provide scientific guidance for Immunodiagnostics and immunotherapy of TB in children (Olin et al., 2018).

Furthermore, children should be closely monitored to ensure that the treatment is effective and to identify any side effects of the medication. Regular follow-up visits should be scheduled and clinical, radiological and laboratory tests should be used to assess the child’s progress and to help and support the family in adhering to the treatment.

## 5 Discussion

The serious situation of childhood tuberculosis has received more attention. The World Health Organization (WHO) has listed “control of tuberculosis in children” as a priority for infectious disease prevention and control, and proposed the goal of “aiming for zero deaths from tuberculosis in children”.

Diagnosing TB in children is challenging. Obtaining samples from children for diagnosis is difficult because they often have fewer bacilli in their sputum. Alternative samples such as blood, urine, cerebrospinal fluid (CSF), feces and breath are being investigated. Corresponding diagnostic tools such as metabolomics and mass spectrometry, especially GC-TOF MS, for clinical testing of MDR/RR-TB are being developed. These methods are not currently recommended for clinical testing due to their limitations, but show promise for future use.

When treating MDR/RR-TB in children, it is important to consider the differences between adults and children when using adult therapies. This includes taking into account age, weight, medical history, drug allergies or sensitivities, and other factors that may affect a child’s response to treatment. In addition, different approaches may be needed to treat MDR-TB in younger and older children due to their different levels of maturity and understanding.

The developing interventions to selectively enhance children’s immunity is urgently needed and the need for a deeper understanding of children’s immunity to identify barriers to early vaccinations and ultimately define new approaches to overcome TB.

Overall, effective management and follow-up during the treatment of drug-resistant TB in children is crucial to ensure the success of the treatment and to minimize the risk of adverse effects.

## Author contributions

KM conceived and designed the article, while KM and XZ contributed to the initial drafting, editing, and supervision of the project. KM and LS secured funding for the study. ZZ and LS led the initial drafting of the manuscript, while XS, HZ, and LL were involved in the critical review and editing of the manuscript. All authors have approved the final version of the article for publication.
